# Global Lifetime and 12-Month Prevalence of Suicidal Behavior, Deliberate Self-Harm and Non-Suicidal Self-Injury in Children and Adolescents between 1989 and 2018: A Meta-Analysis

**DOI:** 10.3390/ijerph16224581

**Published:** 2019-11-19

**Authors:** Kim-San Lim, Celine H. Wong, Roger S. McIntyre, Jiayun Wang, Zhisong Zhang, Bach X. Tran, Wanqiu Tan, Cyrus S. Ho, Roger C. Ho

**Affiliations:** 1 Department of Psychological Medicine, Yong Loo Lin School of Medicine, National University of Singapore, Singapore 119228, Singapore; a0126788@u.nus.edu (K.-S.L.); pcmrhcm@nus.edu.sg (R.C.H.); 2 Division of Child and Adolescent Psychiatry, Department of Psychological Medicine, National University Health System, Singapore 119228, Singapore; celine_hj_wong@nuhs.edu.sg; 3 Mood Disorders Psychopharmacology Unit, University Health Network, University of Toronto, Toronto, ON M5T 1R8, Canada; roger.mcintyre@uhn.ca; 4 Department of Psychiatry, University of Toronto, Toronto, ON M5T 1R8, Canada; 5 Institute of Cognitive Neuroscience, Huaibei Normal University, Huaibei 235000, China; rsczzs@chnu.edu.cn; 6 Institute for Preventive Medicine and Public Health, Hanoi Medical University, Hanoi 100000, Vietnam; bach.ipmph@gmail.com; 7 Johns Hopkins Bloomberg School of Public Health, Johns Hopkins University, Baltimore, MD 21205, USA; 8 Vietnam Young Physicians’ Association, Hanoi 100000, Vietnam; 9 The China-Singapore (Chongqing) Demonstration Initiative on Strategic Connectivity Think Tank, Chongqing 400043, China; cjytwq@163.com; 10 Department of Psychological Medicine, National University Health System, Singapore 119228, Singapore; su_hui_ho@nuhs.edu.sg; 11 Health Innovation and Technology (iHealthtech), National University of Singapore, Singapore 119228, Singapore; 12 Center of Excellence in Behavioral Medicine, Nguyen Tat Thanh University, Ho Chi Minh City 70000, Vietnam

**Keywords:** adolescents, children, meta-analysis, non-suicidal self-injury, deliberate self-harm, suicide

## Abstract

Objective: This meta-analysis aimed to estimate the global lifetime and 12-month prevalence of suicidal behavior, deliberate self-harm and non-suicidal self-injury in children and adolescents. Methods: A systematic search for relevant articles published between 1989 to 2018 was performed in multiple electronic databases. The aggregate 12-month and lifetime prevalence of suicidal behavior, deliberate self-harm, and non-suicidal self-injury were calculated based on the random-effects model. Subgroup analyses were performed to compare the prevalence according to school attendance and geographical regions. *Results*: A total of 686,672 children and adolescents were included. The aggregate lifetime and 12-month prevalence of suicide attempts was 6% (95% CI: 4.7–7.7%) and 4.5% (95% CI: 3.4–5.9%) respectively. The aggregate lifetime and 12-month prevalence of suicidal plan was 9.9% (95% CI: 5.5–17%) and 7.5% (95% CI: 4.5–12.1%) respectively. The aggregate lifetime and 12-month prevalence of suicidal ideation was 18% (95% CI: 14.2–22.7%) and 14.2% (95% CI: 11.6–17.3%) respectively. The aggregate lifetime and 12-month prevalence of non-suicidal self-injury was 22.1% (95% CI: 16.9–28.4%) and 19.5% (95% CI: 13.3–27.6%) respectively. The aggregate lifetime and 12-month prevalence of deliberate self-harm was 13.7% (95% CI: 11.0–17.0%) and 14.2% (95% CI: 10.1–19.5%) respectively. Subgroup analyses showed that full-time school attendance, non-Western countries, low and middle-income countries, and geographical locations might contribute to the higher aggregate prevalence of suicidal behaviors, deliberate self-harm, and non-suicidal self-injury. *Conclusions*: This meta-analysis found that non-suicidal self-injury, suicidal ideation, and deliberate self-harm were the three most common suicidal and self-harm behaviors in children and adolescents.

## 1. Introduction

During the past 30 years, suicide has become a severe cause of mortality across all ages in the world. In 2015, the number of suicide deaths worldwide was estimated to be 788,000 [1], with a global average of 10.7 per 100,000. Suicide was ranked second as a cause of mortality amongst those aged 15–29 years old globally [2], making it a global public health concern. Suicidal behavior, deliberate self-harm and non-suicidal self-injury are important antecedents of suicide in children and adolescents [2]. Suicidal behaviors involve suicidal ideation, planning for suicide and suicide attempts [3]. Self-harm behavior is defined here as an act of intentionally causing harm to own self, irrespective of the type, motive or suicidal intent [2]. Non-suicidal self-injury is defined as deliberate direct destruction or alteration of body tissue without conscious suicidal intent [4]. Deliberate self-harm is an encompassing term for self-injurious behavior, both with and without suicidal intent that has a non-fatal outcome [5]. Non-suicidal self-injury and deliberate self-harm are common in young people who will have borderline personality traits or disorder [6]. Non-suicidal self-injury and deliberate self-harm have been known to predict future suicide attempts [7].

There are potential factors that affect the global prevalence of suicidal and self-harm behavior in children and adolescents. From cross-cultural perspectives, there are ethnic differences in risk factors of suicide attempts [8,9]. In Western countries like Canada, suicide accounts for 10% of deaths in children aged 10 to 14 years and for 23% of deaths in adolescents aged 15 to 19 years [10]. In New Zealand, children and adolescents from the lowest socio-economic status were found to be 31 times more likely to attempt suicide compared to individuals in the higher socio-economic status [11]. In Asia, relationship issues, academic and environmental stressors are common precipitants for suicide attempts among young people [12]. In Singapore, a peak in suicide attempts has been observed in adolescents and young adults aged 15 to 24 years old [12]. Cross-cultural studies found that self-harm behaviors amongst eighth-graders in Hong Kong (23.5%) were less frequent compared to those in the United States (32%) [13]. The lower self-harm rate in Hong Kong adolescents was attributed to cultural differences between the Eastern and Western cultures, with a stronger emphasis on family structures and rules in Asian culture [13]. A meta-analysis is required to study cross-cultural perspectives of suicidal behavior, deliberate self-harm, and non-suicidal self-injury among young people in different countries in a systematic manner.

From a gender perspective, Lewinsohn et al. found female adolescents to have a significantly higher risk of suicide attempts compared to male counterparts, with the differences between genders diminishing as participants increased with age [14]. Furthermore, gender was found to predict lethality in suicide attempts as more males than females made attempts with high perceived lethality and medical lethality [15]. Youth who experienced difficulty in school were at risk for suicide [16]. However, there is little published information specific to the relationship between school attendance, suicidal behaviors, deliberate self-harm, and non-suicidal self-injury in children and adolescents.

Despite the seriousness and scope of the problem, little is known about the global prevalence of suicidal and self-harm behaviors in children and adolescents in the past 30 years. Further research is required to compare the prevalence of suicidal, and self-harm behavior among children and adolescents from different geographical regions as contextual differences (e.g., exposure to adversity) across countries may affect prevalence estimates. Given the above findings and observations, we aimed to conduct a meta-analyze to estimate the global 12-month and lifetime prevalence of adolescents having a history of suicide attempts, suicide plans, suicidal ideation, non-suicidal self-injury, and deliberate self-harm between 1989 to 2018.

## 2. Methods

### 2.1. Search Strategy

During the past thirty years, the advent of computer technology, the Internet, and the widespread use of social media have affected suicidal behaviors, deliberate self-harm and non-suicidal self-injury in young people. Many young people report that computers and the Internet facilitate their communication with peers [17]. The effect of social media on suicidal behaviors, deliberate self-harm and non-suicidal self-injury is still under evaluation. Some studies suggested that social media use has led to the growth of suicide clusters [18], while others showed that it had a positive impact on the prevention of suicide given the myriad of support platforms for the children and adolescents at risk [19]. Given the above findings and observations, this meta-analysis focused from 1989 to 2018.

This study was conducted following the Preferred Reporting Items for Systematic Reviews and Meta-Analyses PRISMA) guidelines. A systematic search was performed with dates covered from 1 January 1989 to 31 December 2018, using a combination of search terms (* indicates truncation): ‘suicid */suicide attempt *’, ‘self harm’ or ’self-harm’,’self injury’ or ’self-injury’, ‘adolescent’, ’youth’, ’young’, ’child *’,’teen *’,’student *’,’school *’ and ‘prevalence’. Electronic databases such as PubMed, Web of Science, PsycINFO, and Embase were utilized. The reference lists of reviews, reports, and other relevant articles were also examined to identify additional studies.

### 2.2. Eligibility Criteria

Two authors (K.-S.L. and R.C.H.) independently identified the eligibility of studies. The studies included in this review must fulfil the following inclusion criteria: (1) the study provided cross-sectional data on the lifetime and 12-month prevalence of suicidal behavior, deliberate self-harm or non-suicidal self-injury; (2) the study population was children or adolescents and (3) a clear definition of suicidal behavior, deliberate self-harm or non-suicidal self-injury were reported. Any study that did not meet the aforementioned inclusion criteria were excluded. Any discrepancies between the two authors were reviewed by another author (C.S.H.) and resolved with consensus.

### 2.3. Data Extraction

Two authors (K.-S.L. and R.C.H.) independently extracted the following data from each eligible study: first author, year of publication, the country where the study was conducted, number of participants with suicidal behavior, deliberate self-harm or non-suicidal self-injury, total sample size, mean age of participants, proportion of female gender and school attendance. Any disagreements between the two authors were resolved via discussion with a third author (C.S.H.). The three authors involved in this process were trained in medicine and psychiatry.

### 2.4. Study Outcomes

A suicide attempt is defined as an act in which an adolescent tries to end his or her life but survives [20]. A suicide plan is a proposed plan of carrying out a suicidal act that may lead to potential death [21]. Suicidal ideation is defined as any self-reported thoughts of engaging in suicide-related behavior [22]. Non-suicidal self-injury is defined as the deliberate, self-inflicted destruction of body tissue without suicidal intent and for purposes not socially sanctioned, such as cutting, burning, and biting [23]. Deliberate self-harm is defined as self-injurious behaviors with and without suicidal intent and that have non-fatal outcomes. The 12-month and lifetime prevalence of suicide attempts, suicide plans, suicide ideation, non-suicidal self-injury, and deliberate self-harm were extracted from each study, which met inclusion criteria.

### 2.5. Statistical Analysis

All statistical analyses were conducted in Comprehensive Meta-analysis statistical software version 3.0 (BioStat Solutions, Inc, Frederick, MD, USA). The aggregate prevalence was calculated based on the random-effects model. The random-effects model was used as it assumes varying effect sizes between studies, because of differing study design and study population [24,25]. A forest plot was then constructed and reported the aggregate prevalence, 95% confidence interval (CI) and *p*-value based on the method adopted by previous meta-analysis on prevalence [26,27]. The statistical significance level was set at *p* < 0.05. The $I^{2}$ statistic was used to assess the between-study heterogeneity [28], which describes the percentage of variance on the basis of real differences in study effects. $I^{2}$ value of 25% was considered low, 50% moderate and 75% substantial [29].

Publication bias was assessed with the utilization of Egger’s regression [25]. A *p*-values of 0.05 or less was used as the cut off for the presence of statistically significant publication bias [30]. The presence of publication bias was then further investigated using both the standard and Orwin’s fail-safe N tests to provide an estimated number of additional studies required to make the eventual effect size insignificant [31]. Meta-regression analyses with a mixed-effect model were performed to identify the effects of potential moderators on the overall heterogeneity. Potential moderators include mean age of sample and proportion of female gender. Subgroup analyses were performed to compare the aggregate prevalence of each study outcome with regards to school attendance and study location. The definitions of developing and developed countries were based on Standard Country or Area Codes for Statistical Use developed by the United Nations [32].

## 3. Results

### 3.1. Selection Results and Study Characteristics

**Figure 1:**
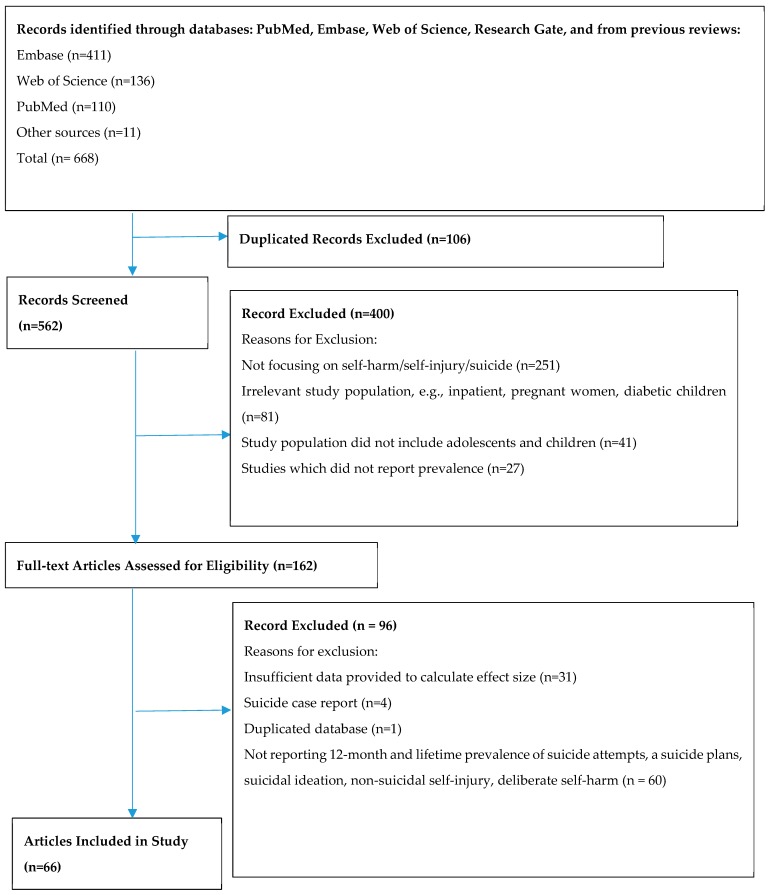
Process of systematic selection using the Preferred Reporting Items for Systematic Reviews and Meta-Analyses flow chart.

A total of 668 potentially relevant citations were gathered after an extensive literature search was performed on the databases listed in Figure 1. A total of 106 studies were found to be duplicated. Of the remaining 562 studies for which titles and abstracts were screened, 400 were excluded. The final 162 studies were then reviewed in full, of which 96 were excluded, leaving 66 studies that met the inclusion criteria to be used in this meta-analysis. The Preferred Reporting Items for Systematic Reviews and Meta-Analyses—chart depicting the detailed process of paper selection can be seen in Figure 1. The 66 studies included in the meta-analysis yields a total population of 686,672 study participants. Table 1 shows the characteristics of the included studies.

### 3.2. Aggregate Prevalence of Suicide Attempts in Children and Adolescents

**Figure 2:**
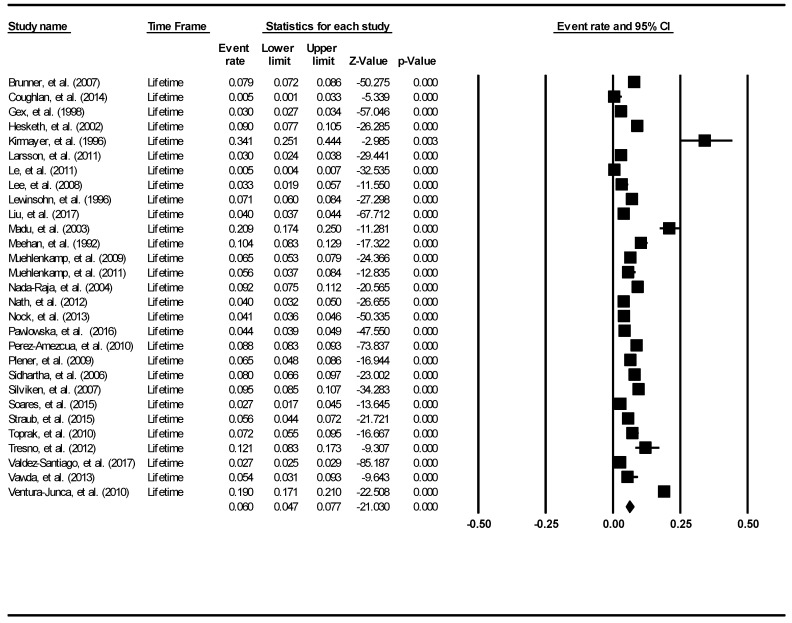
Forest plot of the aggregate lifetime prevalence of suicide attempts.

The aggregate lifetime prevalence of suicide attempts was found to be 6.0% (95% Confidence Interval (CI): 4.7–7.7%). The forest plot is shown in Figure 2. There was a significantly high level of heterogeneity across the included studies (I^2^ = 98.60, *p* <0.001). There was no evidence of publication bias (intercept = 0.16, 95% CI: −5.87–6.2, t = 0.06, df = 27, *p* = 0.96).

**Figure 3:**
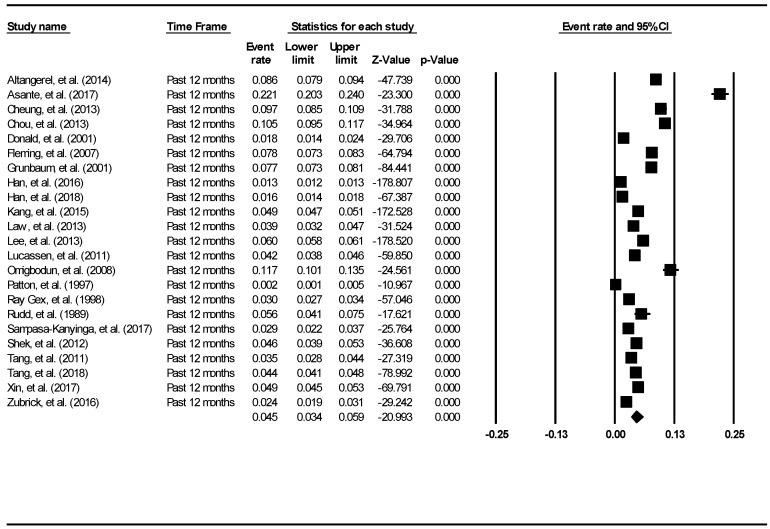
Forest plot of the aggregate 12-month prevalence of suicide attempts.

The aggregate 12-month prevalence of suicide attempts was found to be 4.5% (95% CI: 3.4–5.9%). The result is demonstrated using the forest plot as shown in Figure 3. There was a significant high level of heterogeneity across the included studies (I^2^ = 99.64, *p* < 0.001). There was no evidence of publication bias (intercept = 0.39, 95% CI: −11.71–12.49, t = 0.07, df = 21, *p* = 0.95).

### 3.3. Aggregate Prevalence of Suicide Plans in Children and Adolescents

**Figure 4:**
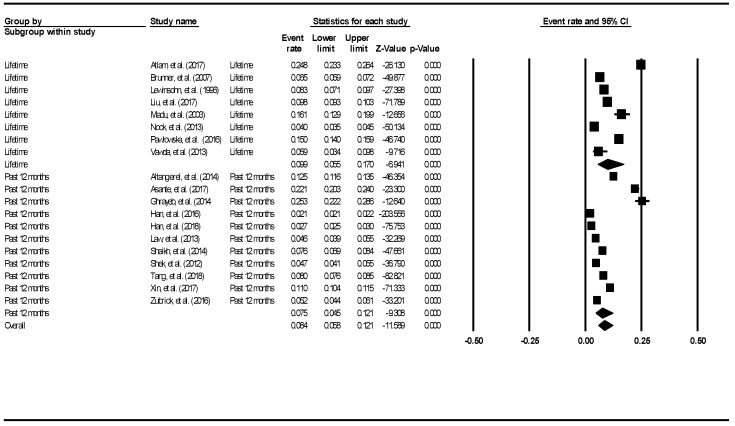
Forest plot of the aggregate lifetime and 12-month prevalence of suicidal plans.

The aggregate lifetime prevalence of suicide plans was found to be 9.9% (95% CI: 5.5–17.0%). The result is demonstrated using the forest plot, as shown in Figure 4. There was a significantly high level of heterogeneity across the included studies (I^2^ = 99.35, *p* < 0.001). The aggregate 12-month prevalence of suicide plans was found to be 7.5% (95% CI: 4.5–12.1%). There was a significantly high level of heterogeneity across the included studies (I^2^ = 99.82, *p* < 0.001). The result is demonstrated using the forest plot, as shown in Figure 4. There was no evidence of publication bias (intercept = 15.24, 95% CI: −5.06–35.54, t = 1.58, df = 17, *p* = 0.13).

### 3.4. Aggregate Prevalence of Suicide Ideation in Children and Adolescents

**Figure 5:**
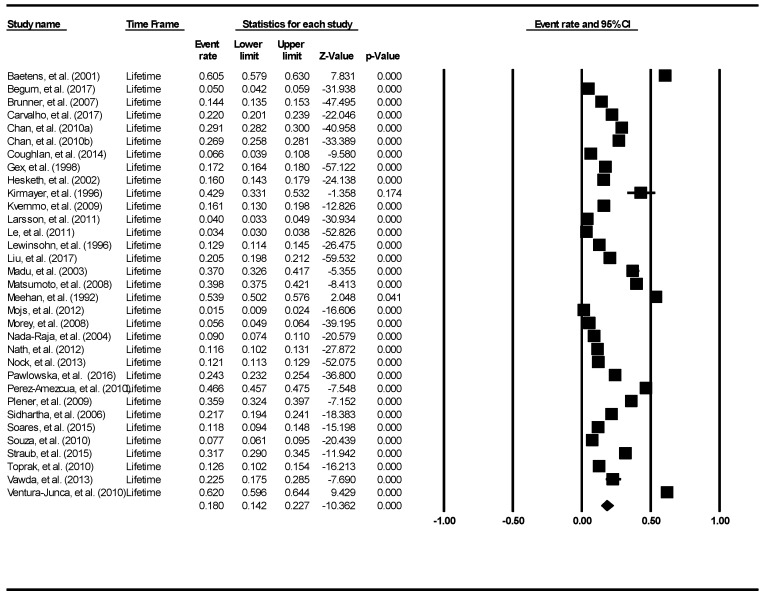
Forest plot of the lifetime aggregate prevalence of suicidal ideation.

The aggregate lifetime prevalence of suicidal ideation was found to be 18% (95% CI: 14.2–22.7%). The result is demonstrated using the forest plot, as shown in Figure 5. There was a significantly high level of heterogeneity across the included studies (I^2^ = 99.68, *p* <0.001). There was no evidence of publication bias (intercept = −11.18, 95% CI: −21.49–0.88, t = 2.21, df = 31, *p* = 0.03).

**Figure 6:**
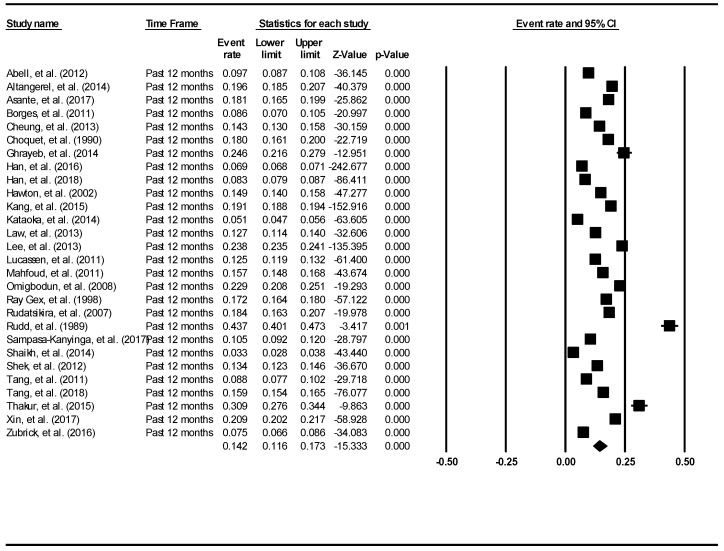
Forest plot of the aggregate 12-month prevalence of suicidal ideation.

The aggregate 12-month prevalence of suicidal ideation was found to be 14.2% (95% CI: 11.6–17.3%). The result is demonstrated using the forest plot as shown in Figure 6. There was a significant high level of heterogeneity across the included studies (I^2^ = 99.82, *p* < 0.001). There was no evidence of publication bias (intercept = −5.18, 95% CI: −18.64–8.29, t = 0.79, df = 26, *p* = 0.44).

### 3.5. Aggregate prevalence of Non-Suicidal Self Injury in Children and Adolescents

**Figure 7:**
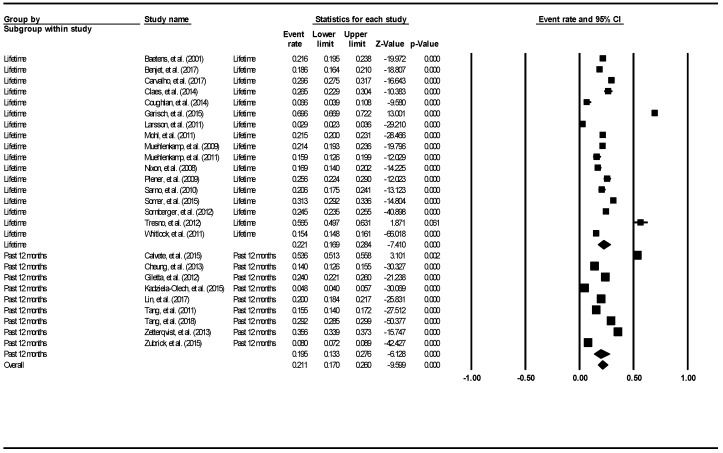
Forest plot of the aggregate lifetime and 12-month prevalence of non-suicidal self-injury.

The aggregate lifetime prevalence of non-suicidal self-injury was 22.1% (95% CI: 16.9–28.4%). The result is demonstrated using the forest plot, as shown in Figure 7. There was a significantly high level of heterogeneity across the included studies (I^2^ = 99.22, *p* < 0.001). The aggregate 12-month prevalence was 19.5% (95% CI: 13.3–27.6%). There was a significantly high level of heterogeneity across the included studies (I^2^ = 99.63, *p* < 0.001). There was no evidence of publication bias (intercept = −4.84, 95% CI: −14.85–6.174, t = 1.0, df = 24, *p* = 0.33).

### 3.6. Aggregate Prevalence of Deliberate Self-Harm in Children and Adolescents

**Figure 8:**
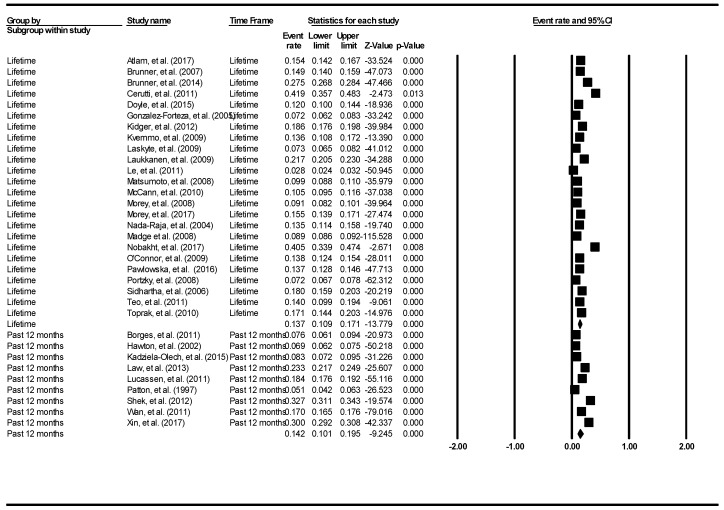
Forest plot of the aggregate lifetime and 12-month prevalence of deliberate self-harm.

The aggregate lifetime prevalence of deliberate self-harm was 13.7% (95% CI: 10.9–17.1%). The result is demonstrated using the forest plot, as shown in Figure 8.

There was a significantly high level of heterogeneity across the included studies (I^2^ = 99.46, *p* < 0.001). The aggregate 12-month prevalence of deliberate self-harm was 14.2% (95% CI: 10.1–19.5%). There was a significantly high level of heterogeneity across the included studies (I^2^ = 99.63, *p* < 0.001). There was no evidence of publication bias (intercept = −9.21, 95% CI: −18.93–0.52, t = 1.93, df = 31, *p* = 0.06).

### 3.7. Subgroup Analyses Based on School Attendance

A higher aggregate lifetime prevalence of suicide attempts is found amongst young people who attended school full-time as compared to young people from the mixed group of education consisting partial and non-school attendees (6.7% (95% CI: 5.3–8.4%) vs. 4.3% (95% CI: 2.7–6.7%)). The aggregate prevalence of suicide attempts in the past 12 months for full-time school attendees (5.6%, 95% CI: 4.2–7.3%) was found to be higher than partial and non-school attendees (2.1%, 95% CI: 1.3–3.6%).

The aggregate lifetime prevalence of suicide plans was found to be 12.4% (95% CI: 8.5–17.8%) in young people who attended school as compared to 5.1% (95% CI: 2.6–9.9%) from partial and non-school attendees. Similarly, the aggregate 12-month prevalence of suicide plans was also higher in the school-attending group (10.3%, 95% CI: 7.6–13.7%) as compared to partial and non-school attending group (3.1%, 95% CI: 1.8–5.2%).

The aggregate lifetime prevalence of suicidal ideation and 12-month prevalence of suicidal ideation were found to be higher in the school-attending group as compared to the partial and non-school attending group (19.5%, 95% CI: 15.0–25.0% vs. 13.9%, 95% CI: 8.5–22.1%) and (14.6%, 95% CI: 11.8–18.0% vs. 12.4%, 95% CI: 7.7–19.5%) respectively.

Both the lifetime and 12-month aggregate prevalence of non-suicidal self-injury were higher in the school-attending group as compared to the partial and non-school attending group (22.8%, 95% CI: 17.1–29.8% vs. 19.0%, 95% CI: 9.7–33.7%), and (21.5%, 95% CI: 15.0–30.0% vs. 8.0%, 95% CI: 2.4–23.3%) respectively.

There was a higher aggregate lifetime and 12-month prevalence of deliberate self-harm in the school-attending group (15.3%, 95% CI: 11.7–19.9%) compared to the partial and non-school attending group (10.4%, 95% CI: 6.6–15.9%).

### 3.8. Subgroup Analyses Based on Western and Non-Western Countries

The aggregate lifetime prevalence of suicide attempts was higher in Western (6.5%, 95% CI: 4.7–9.0%) than non-Western countries (5.4%, 95% CI: 3.6–7.9%). In contrast, aggregate prevalence of suicide attempts in the past 12 months was higher in non-Western countries (6.9%, 95% CI: 4.8–9.6%) than western (2.8%, 95% CI: 1.9–4.0%).

The aggregate lifetime prevalence of suicide plans was higher in non-Western (12.9%, 95% CI: 6.7–23.3%) than Western countries (7.6%, 95% CI: 3.9–14.3%). Similarly, the aggregate past 12-month prevalence of suicide plans was higher in non-Western (10.3%, 95% CI: 7.6–13.7%) than Western countries (3.1%, 95% CI: 1.8–5.2%).

The aggregate lifetime prevalence of suicide ideation was higher in non-Western (18.7%, 95% CI: 12.5–26.9%) than Western countries (17.6%, 95% CI: 12.7–23.8%). The aggregate 12-month prevalence of SI was higher in non-Western (15.2%, 95% CI: 12.6–18.1%) than Western countries (13.0%, 95% CI: 10.5–16.1%).

The aggregate lifetime prevalence of non-suicidal self-injury was higher in non-Western countries (32.6%, 95% CI: 20.0–48.5%) than Western countries (19.4%, 95% CI: 14.2–25.8%). The aggregate 12-month prevalence of non-suicidal self-injury was similar between in non-Western (19.1%, 95% CI: 9.3–35.3%) and the Western countries (19.7%, 95% CI: 10.4–34.2%).

The aggregate lifetime prevalence of deliberate self-harm was higher in Western countries (14.2%, 95% CI: 10.7–18.6%) than non-Western countries (12.8%, 95% CI: 8.5–18.7%). The aggregate 12-month prevalence of deliberate self-harm was higher in non-Western countries (25.2%, 95% CI: 16.8–36.0%). than Western countries (8.5%, 95% CI: 5.5–12.8%).

### 3.9. Subgroup Analyses Based on Developing and Developed Countries

The lifetime prevalence of suicide attempts in developed (6.1% 95% CI: 4.3–8.5%) and low and middle-income countries (6.0% 95% CI: 4.1–7.7%) were similar. However, the past 12-month prevalence of suicide attempts was higher in low and middle-income countries (6.9% 95% CI: 4.8–9.6%) than developed countries (2.8% 95% CI: 1.9–4.0%).

The lifetime prevalence of suicide plans was higher in developing (12.9% 95% CI: 6.7–23.3%) than developed countries (7.6% 95% CI: 3.9–14.3%). Similarly, the 12-month prevalence of suicide plans was higher in low and middle-income countries (10.3% 95% CI: 7.6–13.7%) than developed countries (3.1% 95% CI: 1.8–5.2%).

The lifetime prevalence of suicide ideation was higher in developing (17.7% 95% CI: 11.1–27.0%) than developed countries (17.3% 95% CI: 12.0–24.4%). The 12-month prevalence of suicide ideation was higher in low and middle-income countries (15.9% 95% CI: 13.5–18.6%) than developed countries (11.9% 95% CI: 9.6–14.7%).

The lifetime prevalence of non-suicidal self-injury was significantly higher in low- and middle-income countries (33.7% 95% CI: 19.0–52.5%) as compared to developed countries (20.0% 95% CI: 14.9–26.4%). However, the 12-month prevalence of non-suicidal self-injury was found to be similar between developed countries (19.7% 95% CI: 10.4–34.2%) and low- and middle-income countries (19.1% 95% CI: 9.3–35.3%).

The lifetime prevalence of deliberate self-harm was similar between low- and middle-income countries (13.9% 95% CI: 10.6–18.1%) and developed countries (13.2% 95% CI: 8.5–19.9%). The past 12-month prevalence of deliberate self-harm was found to be higher in low- and middle-income countries (25.2% 95% CI: 16.8–36.0%) than developed countries (8.5 % 95% CI: 5.5–12.8%).

### 3.10. Subgroup Analyses Based on Continents

The lifetime prevalence of suicide attempts was found to be highest in South America (19.0% 95% CI: 17.1–21.0%). The lifetime prevalence of suicide attempts in Africa was 11.2% (95% CI: 2.7%–36.1%). The lifetime prevalence of suicide attempts in Australia was 9.2% (95% CI: 7.5%–11.2%). The lifetime prevalence of suicide attempts in North America was 8.6% (95% CI: 5.4–13.6%). The lifetime prevalence of suicide attempts was lowest in Asia 4.6% (95% CI: 2.7–7.6%) and Europe 4.6% (95% CI: 3.2–6.6%).

The past 12-month prevalence of suicide attempts was found to be highest in Africa at (16.3% 95% CI: 8.4–29%). The past 12-month prevalence of suicide attempts in Asia was 5.8% (95% CI: 4.9–6.7%). The past 12-month prevalence of suicide attempts in Europe was 3% (95% CI: 2.7–3.4%). The past 12-month prevalence of suicide attempts in North America was 3% (95% CI: 1.1–8%). The past 12-month prevalence of suicide attempts was lowest in Australia (2.4%, 95% CI: 1.4–4.4%).

For the lifetime and 12-month prevalence for suicide plans, Asia had the highest prevalence (10.4% 95% CI: 7.7–13.9%). The lifetime and 12-month prevalence of suicide plans in Africa was 13.9% (95% CI: 8.1–22.8%). The lifetime and 12-month prevalence of suicide plans in Europe was 10% (95% CI: 4.3%–21.6%). The lifetime and 12-month prevalence of suicide plans in Australia was 5.2% (95% CI: 4.4–6.1%). The lifetime and 12-month prevalence of suicide plans were lowest in North America (3.7%, 95% CI: 2.3–5.9%).

The lifetime prevalence of suicide ideation was found to be highest in Africa (37.0%, 95% CI: 32.6–41.7%). The lifetime prevalence of suicide ideation in North America was 30.2% (95% CI: 13.4–54.8%). The lifetime prevalence of suicide ideation in South America was 28.5% (95% CI: 8.8–62.3%). The lifetime prevalence of suicide ideation in Asia was 14.2% (95% CI: 8.5–22.7%). The lifetime prevalence of suicide ideation was lowest in Europe (13.7% 95% CI: 9–20.2%).

The past 12-month prevalence of suicide ideation was found to be highest in Africa months (20.6%, 95% CI: 13.7–29.7%). The past 12-month prevalence of suicide ideation in South America was 18.4% (95% CI: 16.3–20.7%). The past 12-month prevalence of suicide ideation in Europe was 16.3% (95% CI: 15.3–17.5%). The past 12-month prevalence of suicide ideation in North America was 12.8% (95% CI: 6.4–24.1%). The lifetime prevalence of suicide ideation was lowest in Asia (13.3%, 95% CI: 10.9–16.3%).

The lifetime and past 12-month prevalence of non-suicidal self-injury were found to be highest in Australia (30.9%, 95% CI: 1.8–91.7%). The lifetime and past 12-month prevalence of non-suicidal self-injury in Asia was 25.7% (95% CI: 18.9–33.8%). The lifetime and past 12-month prevalence of non-suicidal self-injury in North America was 18.7% (95% CI: 14.3–24%). The lifetime and past 12-month prevalence of non-suicidal self-injury were lowest in Europe (18.4%, 95% CI: 12.1–27.2%).

The lifetime and past 12-month prevalence of deliberate self-harm was found to be highest in Asia (17.4%, 95% CI: 12.5–23.7%). The lifetime and past 12-month prevalence of deliberate self-harm in Europe was 12.9% (95% CI: 10.3–16.0%). The lifetime and past 12-month prevalence of deliberate self-harm in Australia was 11.1% (95% CI: 5.4–21.3%). The lifetime and past 12-month prevalence of deliberate self-harm was lowest in North America (7.3%, 95% CI: 6.5–8.2%).

### 3.11. Meta-Regression Analyses

For suicide attempts, mean age (B = 0.0812, z = 2.12, *p* = 0.034) was identified as significant moderator that contributed to heterogeneity between studies. For suicidal plan, mean age (B = 0.20, z = 5.63, *p* < 0.001) was identified as significant moderator that contributed to heterogeneity between studies. For SI, mean age (B = −0.0087, z = −0.28, *p* = 0.78) was a non-significant moderator. For non-suicidal self-injury, mean age (B = 0.11, z = 1.77, *p* = 0.08). was a non-significant moderator. Fordeliberate self-harm, mean age (B = 0.01, z = 0.33, *p* = 0.74) was a non-significant moderator.

For suicide attempts, the proportion of females (B = 1.86, z = 1.05, *p* = 0.29) was a non-significant moderator. For suicidal plan, the proportion of females (B = −0.36, z = −0.14, *p* = 0.89) was a non-significant moderator. For suicidal ideation, the proportion of females (B = 0.77, z = 0.63 *p* = 0.53) was a non-significant moderator. For non-suicidal self-injury, the proportion of females (B = −0.29, z = −0.25, *p* = 0.81) was a non-significant moderator. For deliberate self-harm, the proportion of females (B = −1.79, z = −0.85, *p* = 0.4) was a non-significant moderator.

## 4. Discussion

To the best of our knowledge, this is the first meta-analysis that analyzed suicidal and self-harm phenomena based on 686,672 young people worldwide. The key findings are summarized as follows. non-suicidal self-injury was most frequent with aggregate lifetime and 12-month prevalence of 22.1% and 19.5% respectively. Suicidal ideation was second most frequent with aggregate lifetime and 12-month prevalence of 18% and 14.2% respectively. Deliberate self-harm was third most frequent with aggregate lifetime and 12-montnh prevalence of 13.7% and 14.2% respectively. Suicidal plan ranked fourth with aggregate lifetime and 12-month prevalence 9.9% and 7.5% respectively. Suicide attempt was least frequent with aggregate lifetime and 12-month prevalence of 6.0% and 4.5% respectively.

This meta-analysis found that the aggregate lifetime prevalence of suicide attempts was higher in Western (6.5%, 95% CI: 4.7–9.0%) than non-Western countries. There are several reasons to explain higher prevalence of suicide attempts among young people in western countries. First, substance abuse appeared to have affected suicide rates of young males in Western countries [129]. Second, high suicide rates among young indigenous people in Western countries have been attributed to internalised anger and despair related to social disruption and disempowerment [130]. Third, young people in Western countries could have more access to suicide means, including firearms. In contrast, the aggregate lifetime prevalence of suicide plans, suicide ideation and non-suicidal self-injury were higher in non-Western countries than Western countries. This finding suggests that young people in non-Western countries could have thought about suicide but did not attempt suicide. Attempted suicide is illegal in some of the non-Western countries including Bangladesh, Hungary, India and Japan, though in Japan it is not punishable [129]. The legal implication could deter suicide attempts in some of the non-Western countries.

This meta-analysis found that non-suicidal self-injury had the highest aggregate lifetime and 12-month prevalence worldwide. Non-suicidal self-injury is defined as the intentional destruction of one’s own body tissue without suicidal intent [27]. Examples of non-suicidal self-injury, including self-laceration, skin scratching, burning and hitting. Klonsky et al. proposed functional theories that explain the reasons for non-suicidal self-injury in young people [131]. The reasons include alleviation of negative emotion, self-punishment, self-directed anger, and expression of distress. Klonsky et al. highlighted the misconception that non-suicidal self-injury is always a symptom of borderline personality disorder [132]. For young people with engaging non-suicidal self-injury, a psychological intervention which aims at building positive emotion, reducing self-directed anger and promoting more adaptive way to express distress may reduce the prevalence of non-suicidal self-injury.

A recent study found that the 12-month prevalence rates of youth self-harm in low and middle- income countries were comparable to high-income countries. This meta-analysis with a larger sample size showed that children and adolescents from low- and middle-income countries with lower income had a higher aggregate 12-month prevalence of deliberate self-harm than children and adolescents from developed countries with higher income. Previous research reported that non-suicidal self-injury appeared to be more common among Caucasians than non-Caucasians [133]. Our meta-analysis found that the 12-month prevalence of non-suicidal self-injury was highest in Australia which has 74.3% of the population who are Caucasians [134].

The subgroup analysis yielded several interesting findings. The aggregate lifetime and 12-month prevalence of suicidal and self-harm behavior were higher in full-time school attendees as compared with partial and non-attendees. School attendees are more likely to be exposed to risk factors, including academic stress and school bullying. Academic stress leads to anger, anxiety, helplessness, shame, and boredom [135]. A previous study found that skin picking, which causes skin damage was positively correlated with academic stress and trait anxiety was a predisposing factor [136]. The other risk factors faced by school attendees are peer victimization, which reflects the experience of overt (e.g., hitting, pushing), reputational (e.g., spreading rumors), or relational aggression from peers (e.g., being excluded, gossiped about) [137,138]. Vergara et al. (2019) found that peer victimization was associated with the frequency of past month non-suicidal self-injury thoughts and past month non-suicidal self-injury behaviors [139]. The aggregate lifetime and 12-month prevalence of suicidal and self-harm behavior were higher in developing and non-western countries.

We found that the lifetime prevalence of suicide ideation, the 12-month prevalence of suicide attempts and suicide ideation were highest in Africa. This could be due to the fact that large numbers of African children and adolescents were exposed to adverse childhood experiences [140]. Young people in developing and non-western countries are more likely to be exposed to adverse childhood experiences including alcohol abuse [141], lack of access to care for mental health problems, orphanage, and early parental death [142], human immunodeficiency virus (HIV) infection [142] and violence against children and adolescents [143]. Interventions to reduce African children and adolescent suicidality include those improving family functioning, reducing poverty, mitigating the impacts of HIV and the provision of effective mental health services for adversity-exposed children and adolescent [140]. As the infrastructure for mental health service is still developing, the main challenge is to reach out to children and adolescents in low and middle-income countries and educate them to handle suicidal and self-harm behaviors. Electronic health (E-health) was found to provide a cost-effective solution in mental health [144]. As the use of smartphones becomes increasingly prevalent and affordable, more children and adolescents in low and middle-income countries can own a smartphone device and download health-related applications [17]. Proof of concept feasibility studies and randomized trials should be conducted in low and middle-income countries to determine that smartphone applications are efficacious to reduce suicidal and self-harm behavior in children and adolescents before their actual implementation [145].

The lifetime and 12-month prevalence of suicide plans and DSH were found to be highest in Asia. Asian children and adolescents face more academic-related stress due to the competitiveness in the education system, and getting poor grades in the examination have been found to bear two major significant sources of anxiety and depression amongst Asian children and adolescents [146]. Willingness to seek help was found to be a protective factor against suicidal and self-harm behavior for Asian children and adolescents [15]. Nevertheless, help-seeking from peers may not be beneficial [147]. Children and adolescents may not receive the help that they require, as peers often might be poorly equipped to provide appropriate advice.

Meta-regression found that age was a critical moderator that explains for heterogeneity for a lifetime and 12-month prevalence of suicide attempts and suicide plans. Children and young adolescents were less exposed to suicide risk factors as compared with older adolescents [148]. Older adolescents are predisposed to specific risk factors associated with suicidal behaviors, including baseline interpersonal problems in one’s social circle [149], psychiatric disorders [148], and STD-related risk [150]. Meta-regression also found that gender was not a vital moderator that explains for heterogeneity of the prevalence of suicidal and self-harm behavior. This finding may challenge the gender paradox, which suggests significant epidemiological differences in suicidal and self-harm behaviors between adolescent females and males [151]. A previous study found that there were no gender differences in family problems and school problems which are well-known risk factors associated with suicidal and self-harm behavior in young people [152].

The strengths of this meta-analysis include an extensive search in identifying a large number of articles on suicidal and self-harm behaviour in 686,672 children and adolescents, adherence to the guidelines, the inclusion of meta-regression and subgroup analysis as well as lack of publication bias [28]. Nevertheless, this meta-analysis has several limitations. First, this meta-analysis classified suicidal and self-harm phenomena into five sub-categories and not able to study their inter-relationship. Klonsky et al. proposed that non-suicidal self-injury may be an essential risk factor for suicidal behaviour [132]. Future study is required to study the inter-relationship between suicidal behaviour, deliberate self-harm, and non-suicidal self-injury in young people. Second, we could not classify dliberate self-harm as suicidal and non-suicidal deliberate self-harm. Future research is required to understand the differences between young people who attempt suicidal and non-suicidal deliberate self-harm.

## 5. Conclusions

In conclusion, this meta-analysis found that the three most common suicidal and self-harm behaviors were non-suicidal self-injury (aggregate lifetime and 12-month prevalence of 22.1% and 19.5% respectively), suicidal ideation (aggregate lifetime and 12-month prevalence of 18% and 14.2% respectively) and deliberate self-harm (aggregate lifetime and 12-month prevalence of 13.7% and 14.2% respectively). The aggregate lifetime prevalence of suicide attempts was higher in Western than non-Western countries, in contrast, the aggregate lifetime prevalence of suicide plans, suicide ideation and non-suicidal self-injury were higher in non-Western countries than Western countries. Suicidal and self-harm behavior was higher in children and adolescents who were full-time school attendees and those who live in developing countries. Meta-regression analyses showed that the mean age of participants was a significant moderator that contributed to heterogeneity for a lifetime and 12-month prevalence of suicide attempts and suicidal plans. Psychological interventions targeting self-harm and suicidal behavior, social interventions targeting adversities in low- and middle-income countries, and electronic—health interventions to reach out to children and adolescents may reduce the global prevalence of suicidal and self-harm behavior in children and adolescents.

## Figures and Tables

**Table 1:** Characteristics of the Studies Included in This Meta-Analysis.

First Author	Year	Study Location	Sample Size	Mean Age	Proportion of Female Gender	Prevalence of Suicide Attempts	Prevalence of Suicide Plans	Prevalence of Suicide Ideation	Prevalence of Non-Suicidal Self-Injury	Prevalence of Deliberate Self-Harm
Abell [33]	2012	Jamaica	2997	NA	NA	NA	NA	12M:0.097	NA	NA
Altangerel [34]	2014	Mongolian	5191	NA	0.567	12M: 0.086	12M:0.125	12M: 0.196	NA	NA
Asante [35]	2017	Ghana	1984	NA	0.458	12M: 0.221	12M:0.221	12M: 0.181	NA	NA
Atlam [36]	2017	Turkey	2973	NA	0.548	NA	LT: 0.248	NA	NA	LT: 0.154
Baetens [37]	2011	Belgium	1417	15.13	0.814	NA	NA	LT: 0.605	LT: 0.216	NA
Begum [38]	2017	Bangladesh	2476	NA	NA	NA	NA	LT:0.05	NA	NA
Benjet [39]	2017	Mexico	1071	NA	0.57	NA	NA	NA	LT: 0.186	NA
Borges [40]	2011	United States	1004	NA	0.56	NA	NA	12M:	NA	12M: 0.076
Brunner [41]	2007	Germany	5759	14.9	0.498	LT: 0.079	LT: 0.065	LT: 0.144	NA	LT: 0.149
Brunner [42]	2014	Various European countries	12073	14.9	0.556	NA	NA	NA	NA	LT:0.275
Calvete [43]	2015	Spain	1864	15.32	0.514	NA	NA	NA	12M: 0.536	NA
Carvalho [44]	2017	Brazil	1763	16.75	0.53	NA	NA	LT: 0.22	LT:0296	NA
Cerutti [45]	2011	Italy	234	16.47	0.5	NA	NA	NA	NA	LT: 0.419
Chan [46]	2008	Hong Kong	10239	NA	NA	NA	NA	LT: 0.291	NA	NA
Chan [46]	2008	Hong Kong	5688	NA	NA	NA	NA	LT: 0.269	NA	NA
Cheung [47]	2013	Hong Kong	2317	16.4	0.548	12M: 0.0967	NA	12M: 0.143	12M: 0.14	NA
Choquet [48]	1990	France	1519	14.7	0.45	NA	NA	12M:0.18	NA	NA
Chou [49]	2013	Taiwan	2835	19.75	0.554	12M: 0.105	NA	NA	NA	NA
Claes [50]	2013	Belgium	532	15.11	0.258	NA	NA	NA	LT: 0.265	NA
Coughlan [51]	2014	Ireland	212	11.54	0.519	LT: 0.005	NA	LT: 0.068	LT: 0.066	0.068
Donald [52]	2001	Australia	3082	NA	NA	LT: 0.0185	NA	NA	NA	NA
Doyle [53]	2015	Ireland	856	NA	0.5	NA	NA	NA	NA	LT: 0.12
Fleming [54]	2007	New Zealand	9570	NA	0.539	12M:0.078	NA	NA	NA	NA
Garisch [55]	2015	New Zealand	1162	16.35	0.615	NA	NA	NA	LT: 0.696	NA
Ghrayeb [56]	2014	Palestine	720	15.4	0.496	NA	12M: 0.253	12M: 0.246	NA	NA
Giletta [57]	2012	Italy, Netherlands, United States	1862	15.69	0.49	NA	NA	NA	12M: 0.24	NA
Gonzalez-Forteza [55]	2005	Mexico	2531	16.67	0.544	0.808	NA	NA	NA	LT: 0.072
Grunbaum [58]	2001	United States	16262	16.16	NA	12M: 0.077	NA	NA	NA	NA
Han [59]	2016	United States	135300	NA	0.498	12M: 0.013	12M:0.0214	12M: 0.069	NA	NA
Han [60]	2018	United States	17000	NA	NA	12M:0.016	12M:0.027	12M: 0.083	NA	NA
Hawton [61]	2002	United Kingdom	5801	NA	0.466	NA	NA	12M: 0.149	NA	12M: 0.069
Hesketh [62]	2002	China	1576	NA	NA	LT: 0.090	NA	LT: 0.160	NA	NA
Kądziela-Olech [63]	2015	Poland	2220	16.7	0.463	NA	NA	NA	12M:0.048	12M:0.083
Kang [64]	2015	South Korea	72623	NA	NA	12M: 0.049	12M: 0.191	12M:0.191	NA	NA
Kataoka [65]	2014	Japan	9778	NA	0.486	NA	NA	12M: 0.05	NA	NA
Kidger [66]	2012	England	4855	16.67	0.589	NA	NA	NA	NA	LT: 0.186
Kvernmo [67]	2009	Norway	447	14.7	0.526	NA	NA	12M:0.161	NA	LT: 0.136
Kirmayer [68]	1996	Canada	99	19.4	0.516	LT: 0.341	NA	LT: 0.429	NA	NA
Larsson [69]	2008	Norway	2464	13.7	0.508	LT: 0.030	NA	LT: 0.040	LT: 0.029	NA
Laskyte [70]	2009	Lithuania	3848	NA	0.572	NA	NA	NA	NA	LT:0.07
Law [13]	2013	Hong Kong	2579	12	0.5	12M: 0.039	NA	12M:0.046	NA	12M:0.233
Laukkanen [71]	2009	Finland	4205	15.58	0.536	NA	NA	NA	NA	LT: 0.217
Le [72]	2011	Vietnam	7584	NA	0.560	LT: 0.005	NA	LT: 0.034	NA	LT: 0.028
Lee [73]	2008	South Korea	368	NA	0.389	LT: 0.033	NA	TW: 0.098	NA	NA
Lee [74]	2013	South Korea	74698	NA	0.472	12M:0.0597	NA	12M:0.238	NA	NA
Lewinsohn [75]	1996	USA	1709	NA	NA	LT: 0.071	LT: 0.083	LT: 0.129	NA	NA
Lin [76]	2017	Taiwan	2170	15.83	0.511	NA	NA	NA	12M:0.2	NA
Liu [77]	2018	China	11831	14.97	0.49	LT: 0.040	LT: 0.098	LT: 0.205	NA	NA
Lucassen [78]	2011	New Zealand	9107	NA	0.46	12M: 0.042	NA	12M:0.125	NA	12M:0.184
Madu [79]	2003	South America	435	17.25	0.559	LT: 0.209	LT: 0.161	LT: 0.371	NA	NA
Mahfoud [80]	2011	Lebanon	5109	13.8	0.543	NA	NA	12M: 0.157	NA	NA
Madge [5]	2008	Australia/Belgium/ England/Hungary/ Ireland/The Netherlands/ Norway.	30427	15.6	0.49	NA	NA	NA	NA	LT: 0.089
Matsumoto [81]	2008	Japan	1726	14.5	0.51	NA	NA	LT: 0.398	NA	LT: 0.099
McCann [82]	2010	Ireland	3178	NA	0.59	NA	NA	NA	NA	LT: 0.105
Meehan [83]	1992	United States	694	NA	NA	LT: 0.104	NA	LT 0.539	NA	NA
Mohl [84]	2011	Denmark	2864	17	0.608	NA	NA	LT: 0.215	NA	NA
Mojs [85]	2012	Poland	1065	NA	0.72	NA	NA	LT: 0.015	NA	NA
Morey [86]	2008	Ireland	3646	16.01	0.53	NA	NA	LT: 0.056	NA	LT: 0.091
Morey [87]	2017	England	2000	15.6	0.52	NA	NA	NA	NA	LT: 0.155
Muehlenkamp [88]	2009	United States	1375	15.48	0.561	LT: 0.065	NA	NA	LT: 0.214	NA
Muehlenkamp [89]	2011	United States	390	16.27	0.549	LT: 0.056	NA	NA	LT 0.159	NA
Nada-Raja [90]	2004	New Zealand	966	NA	0.489	LT: 0.092	NA	LT: 0.090	NA	LT: 0.135
Nath [91]	2012	India	1817	19.11	NA	LT: 0.040	NA	LT: 0.116	NA	NA
Nixon [92]	2008	Canada	568	15.2	0.537	NA	NA	NA	LT: 0.169	NA
Nobakht [93]	2017	Iran	200	NA	0.5	NA	NA	NA	NA	LT: 0.405
Nock [94]	2013	USA	6483	NA	0.482	LT: 0.040	LT: 0.040	LT: 0.121	NA	NA
O’Connor [95]	2009	Scotland	1967	NA	0.534	NA	NA	NA	NA	LT: 0.138
Omigbodun [96]	2008	Nigeria	1429	14.4	0.491	12M: 0.117	NA	12M: 0.229	NA	NA
Patton [97]	1997	Australia	1699	NA	NA	12M: 0.002	NA	NA	NA	12M: 0.051
Pawlowska [98]	2016	Poland	5685	17.18	0.3	LT: 0.044	LT: 0.150	LT: 0.243	NA	LT: 0.137
Pérez-Amezcua [99]	2010	Mexico	12424	NA	0.55	LT: 0.088	NA	LT: 0.466	NA	NA
Plener [100]	2009	USA	665	14.8	0.571	LT: 0.065	NA	LT: 0.359	LT: 0.256	NA
Portzky [101]	2008	Netherlands/Belgium	8889	15.48	0.51	NA	NA	NA	NA	LT: 0.072
Rey Gex [102]	1998	Switzerland	9268	17.46	0.431	LT: 0.030	NA	12M: 0.172	NA	NA
Rudatsikira [103]	2007	Guyana	1197	NA	0.579	NA	NA	12M: 0.184	NA	NA
Rudd [104]	1989	United States	737	NA	0.61	12M: 0.056	NA	12M:0.437	NA	NA
Sampasa-Kanyinga [105]	2017	Canada	1922	14.4	0.54	12M: 0.029	NA	12M: 0.105	NA	NA
Sarno [106]	2010	Italy	578	NA	0.825	NA	NA	NA	LT: 0.206	NA
Shaikh [107]	2014	India	5184	NA	0.248	NA	12M:0.076	12M: 0.033	NA	NA
Shek [108]	2012	Hong Kong	3328	12.59	0.472	NA	12M:0.0475	12M: 0.134	NA	12M: 0.327
Sidhartha [109]	2006	India	1205	14.73	0.4	LT: 0.080	NA	LT: 0.217	NA	LT: 0.180
Silviken [110]	2007	Norway	2691	16.9	0.521	LT: 0.095	NA	SM: 0.151	NA	NA
Soares [111]	2015	Brazil	549	NA	0.801	LT:0.027	NA	LT: 0.118	NA	NA
Somer [112]	2015	Turkey	1656	16.8	0.55	NA	NA	NA	LT: 0.313	NA
Sornberger [113]	2012	Canada	1744	14.92	0.508	NA	NA	NA	LT: 0.245	NA
Straub [114]	2015	Germany	1117	14.83	0.527	LT:0.056	NA	LT: 0.317	NA	NA
Tang [115]	2011	Hong Kong	2013	15.6	0.453	12M: 0.0348	NA	12M:0.088	12M: 0.155	NA
Tang [116]	2018	China	15623	15.2	0.485	12M: 0.0443	12M:0.08	12M: 0.159	12M: 0.292	NA
Teo [117]	2011	Australia	207	NA	NA	NA	NA	NA	NA	LT: 0.14
Thaku [118]	2015	India	705	NA	0.488	NA	NA	12M: 0.309	NA	NA
Toprak [119]	2010	Turkey	636	19.36	0.539	LT:0.072	NA	LT: 0.126	NA	LT: 0.171
Tresno [120]	2012	Indonesia	207	19.78	NA	LT:0.121	NA	NA	LT: 0.565	NA
Valdez-Santiago [121]	2017	Mexico	21509	15.4	NA	LT: 0.027	NA	NA	NA	NA
Vawda [122]	2013	South Africa	222	13.3	0.482	LT: 0.054	LT: 0.059	LT 0.225	NA	NA
Ventura-Junca [123]	2010	Chile	1567	16.2	0.459	LT: 0.190	NA	LT: 0.620	NA	NA
Wan [124]	2011	China	17622	16.1	0.512	NA	NA	NA	NA	12M: 0.17
Whitlock [125]	2011	United States	11529	NA	0.576	NA	NA	NA	LT:0.154	NA
Xin [126]	2017	China	11880	14.62	0.505	12M: 0.0491	12M:0.11	12M: 0.209	NA	12M: 0.30
Zetterqvist [127]	2013	Sweden	3060	NA	0.505	NA	NA	NA	12M:0.356	NA
Zubrick [128]	2016	Australia	2563	NA	0.692	12M: 0.0241	12M: 0.052	12M: 0.075	12M:0.08	NA
